# What matters when managing childhood fever in the emergency department? A discrete-choice experiment comparing the preferences of parents and healthcare professionals in the UK

**DOI:** 10.1136/archdischild-2019-318209

**Published:** 2020-02-27

**Authors:** Simon Leigh, Jude Robinson, Shunmay Yeung, Frans Coenen, Enitan D Carrol, Louis W Niessen

**Affiliations:** 1 Institute of Infection and Global Health, University of Liverpool, Liverpool, UK; 2 School of Social and Political Sciences, University of Glasgow, Glasgow, UK; 3 Department of Clinical Research, MARCH Centre for Maternal, Adolescent, Reproductive and Child Health, LSHTM, London, UK; 4 Department of Computer Science, University of Liverpool, Liverpool, Merseyside, UK; 5 Department of International Public Health, Liverpool School of Tropical Medicine, Liverpool, UK

**Keywords:** infectious diseases, qualitative research, discrete choice experiment, paediatrics, emergency care

## Abstract

**Background:**

Fever among children is a leading cause of emergency department (ED) attendance and a diagnostic conundrum; yet robust quantitative evidence regarding the preferences of parents and healthcare providers (HCPs) for managing fever is scarce.

**Objective:**

To determine parental and HCP preferences for the management of paediatric febrile illness in the ED.

**Setting:**

Ten children’s centres and a children’s ED in England from June 2018 to January 2019.

**Participants:**

98 parents of children aged 0–11 years, and 99 HCPs took part.

**Methods:**

Nine focus-groups and coin-ranking exercises were conducted with parents, and a discrete-choice experiment (DCE) was conducted with both parents and HCPs, which asked respondents to choose their preferred option of several hypothetical management scenarios for paediatric febrile illness, with differing levels of visit time, out-of-pocket costs, antibiotic prescribing, HCP grade and pain/discomfort from investigations.

**Results:**

The mean focus-group size was 4.4 participants (range 3–7), with a mean duration of 27.4 min (range 18–46 min). Response rates to the DCE among parents and HCPs were 94.2% and 98.2%, respectively. Avoiding pain from diagnostics, receiving a faster diagnosis and minimising wait times were major concerns for both parents and HCPs, with parents willing-to-pay £16.89 for every 1 hour reduction in waiting times. Both groups preferred treatment by consultants and nurse practitioners to treatment by doctors in postgraduate training. Parents were willing to trade-off considerable increases in waiting times (24.1 min) to be seen by consultants and to avoid additional pain from diagnostics (45.6 min). Reducing antibiotic prescribing was important to HCPs but not parents.

**Conclusions:**

Both parents and HCPs care strongly about reducing visit time, avoiding pain from invasive investigations and receiving diagnostic insights faster when managing paediatric febrile illness. As such, overdue advances in diagnostic capabilities should improve child and carer experience and HCP satisfaction considerably in managing paediatric febrile illness.

**What is already known on this topic?**

- Children with fever account for 10%–20% of emergency department attendances, yet little is known about the preferences of healthcare providers (HCPs) and parents regarding management.
- Diagnosing a definitive cause of fever is often an iterative and protracted process, which may inconvenience both patients and parents, and require significant resources from HCPs.
- Efforts to reduce diagnostic uncertainty are focusing on the development of point-of-care testing; however, evidence regarding preferences, potential uptake and outcomes in emergency care is limited.

**What this study adds?**

- Avoiding pain from diagnostics and minimising time to diagnosis and discharge are major concerns for parents and HCPs when investigating paediatric febrile illness.
- Reducing antimicrobial prescribing is the single largest concern for HCPs. Conversely, parents exhibited no preference for/against antibiotics, contrary to existing evidence.
- Children, carers and HCPs are all likely to benefit considerably from upcoming advances in diagnostics, which are expected to provide increased confidence in timely decision making.

## Introduction

Children with fever account for 14% of emergency department (ED) attendances in England.^1 2^ Though most display signs and symptoms suggestive of specific infections; in ~20% of cases, there is no obvious cause.^3 4^ These children are a concern to healthcare providers (HCPs), due to a small but significant risk of life-threatening bacterial infections,^5^ which can have catastrophic consequences if undetected.

Diagnosing the source of fever is therefore a lengthy process, often including both blood and urine investigations, radiography and in some cases lumbar puncture.^6^ Invasive investigations may inconvenience both patients and parents; and consequently, efforts to reduce diagnostic uncertainty are focusing on the development of protein-based or RNA signatures, delivered via point-of-care (POC) testing. Evidence from primary care suggests that such tests may be effective in preventing clinically unnecessary antibiotic use and empiric investigations;^7–9^ however, evidence in emergency care is lacking, and there is currently little agreement as to whom such tests should be used for.^10 11^

Decisions made during the management of paediatric febrile illness mitigate diagnostic uncertainty and contribute to patient and carer satisfaction with care. Parental anxiety and fear of serious but rare illness, including sepsis,^4^ can result in parents of febrile children expecting antibiotics even when not clinically indicated,^12 13^ while some may prefer their child to be managed by a more experienced clinician.^14 15^ With the development of more sensitive, accurate and faster diagnostics, processes for investigating febrile illness are likely to change. What is unclear, are the expectations of parents and HCPs alike when managing paediatric febrile illness.

We conducted a series of focus-groups and a discrete-choice experiment (DCE) among parents and HCPs, to determine preferences for existing and future paediatric febrile illness care pathways, establishing the likely impact and success of implementing novel diagnostics for the management of paediatric febrile illness.

## Methods

We conducted focus-groups and discrete-choice surveys from June 2018 to January 2019 to determine parental and HCP preferences for the management of paediatric febrile illness. Participants consented in writing after being provided with a participant information sheet and having had the opportunity to ask questions. Demographic information for all respondents was collected immediately following consent.

### Focus group discussions

We followed methodological guidelines from the International Society for Pharmacoeconomics and Outcomes Research,^16^ identifying attributes of potential importance through a literature review, discussion with experts in paediatric infectious diseases, historical observational data^17^ and focus-groups.

Initially, nine focus-groups took place with parents of children aged <11 years, in seven locations across the North-West of England between June and July 2018. The mean group size was 4.4 participants, with a mean duration of 27.4 min. Focus-groups were moderated by the principal researcher, and observed by staff from each venue, who were familiar with the participant groups. Respondents were invited to discuss any theme they considered relevant to the management of fever in children, with a focus on waiting times, preferred HCPs, staying overnight, having many tests, pain from investigations, antibiotics and time waiting to receive updates. Following the focus-groups, respondents were provided with printed labels and 100 coins, and asked to assign the coins to the attributes/labels they believed were most important. The results of this exercise can be found in online supplementary table 1. Following this exercise, the attributes ‘staying overnight’ and ‘having lots of tests’ were removed due to their respective lack of coins allocated. Although receiving antibiotics was the least important to parents, this was not ruled out due to the expected importance to decision making among HCPs. Finally, multicollinearity with ‘time waiting in the ED’, meant the theme ‘time until receiving information/updates’ was replaced with a binary variable of ‘receive POC test’ for the purpose of the DCE.

10.1136/archdischild-2019-318209.supp1 Supplementary data

### Discrete-choice experiment

DCE methodology is well described^18 19^ and used extensively to measure patients’ preferences for healthcare services. In DCEs, respondents are given a hypothetical scenario, typically comparing one option to another, and asked to choose which of the available options they prefer.^18 19^ This process is repeated with the values (levels) of the characteristics (attributes) changing each time. The attributes used for our DCE are listed in table 1, with levels determined from responses obtained during the focus-groups and previously published data from our hospital.^17^ The DCE was provided using paper forms and on a tablet-PC (the full survey is provided in online supplementary figure 1).

10.1136/archdischild-2019-318209.supp2 Supplementary data

**Table 1:** Attributes and levels of the discrete-choice experiment

Attribute	Levels
Healthcare provider treating child	Doctor in postgraduate training* Nurse practitioner Consultant†
Pain experienced from investigations	Low Moderate
Likelihood of receiving antibiotics	Low (7%) Moderate (20%) High (33%)
Total time in the emergency department	1 hour 2 hours 3 hours 4 hours
Out-of-pocket cost to parent/guardian	£7 (~$9) £12 (~$16) £20 (~$26)
Receive rapid point of care test during triage	Yes No

*Consultant (UK) is equivalent to an attending physician in the USA.

†Foundation Year 1 and 2 in UK = Internship (North America and Europe).

There were two groups of respondents: (1) HCPs working in a children’s ED and (2) parents recruited from children’s soft play centres. We consecutively invited parents of children aged 0–11 years and excluded those unable to read/communicate proficiently in English. For HCPs, we included qualified nursing and medical staff of all grades with experience of managing febrile children, working within our tertiary care specialist hospital, located in the North West of England. Each respondent received 14 discrete-choice tasks plus two tests of rationality, one as the first task, to gauge understanding, and one as the final task, to measure sustained concentration. Failing either test of rationality led to responses being excluded from analysis. Respondents chose between two scenarios for managing paediatric febrile illness, characterised by differing levels of the attributes included (online supplementary figure 1). No opt-out option was included as this was deemed unrealistic in emergency care. As the full factorial experiment required (3^3^×2^2^×4^1^=432) choices per respondent, a D-optimal design was chosen, with two blocks, with the order choice tasks were presented randomised using a random number generator. Surveys were pilot tested with 10 parents and 5 HCPs not involved in the main study to gauge interpretation and response times, during which period a researcher was available to answer any questions. Although sample-size calculations represent a technical challenge in DCEs, we used a parametric approach^20^ to determine sample-size, equal to 48 respondents per group.

### Data analysis

We used a mixed-logit model to estimate parental and HCP preferences for the management of paediatric febrile illness. Effects coding was used for all categorical variables; detailed explanations of which are provided in online supplementary materials. To account for heterogeneity in preferences among our sample, including parents having different views on management by nurse practitioners, or doctors having different views on waiting times to nurses, it was assumed that population preferences for all effects-coded variables followed a normal distribution. As such, each individual preference observed constituted a random draw from this population distribution. Waiting times and costs were coded as linear continuous variables. We first estimated a main-effects model, and subsequently estimated subgroup effects, which for parents, were determined from the focus-group exercise, and included variables such as parent age, child age and the number of children a parent had. Due to a lack of qualitative research with HCPs prior to the DCE, subgroup analyses of HCP preferences were determined by the clinical lead for the study. Willingness-to-pay (WTP) and willingness-to-wait (WTW) analyses were performed to determine how respondents were willing to trade off attributes. CIs for WTP and WTW estimates were estimated via joint-distributed bootstrapping. All analyses were performed using Stata 14 (Stata) and deemed statistically significant at the 5% level.

10.1136/archdischild-2019-318209.supp3 Supplementary data

## Results

### Characteristics of participants

Between June 2018 and January 2019, 154 eligible parents and 101 eligible HCPs were identified. Fifty parents were invited to participate in focus-groups, 40 of whom accepted and 24 of which took part in the coin-ranking exercise. The remaining 104 parents and 101 HCPs were invited to take part in the DCE. Two parents and one HCP did not complete the DCE and four parents and one HCP declined to take part, leaving a total of 98 parents and 99 HCPs (online supplementary figure 2). No one failed either of the tests of rationality, resulting in a 100% understanding rate. Tables 2 and 3 illustrate the demographics of those completing the DCE in the parental and HCP cohorts, respectively.

10.1136/archdischild-2019-318209.supp4 Supplementary data

**Table 2:** Characteristics of parents

Percentage	Number
*Characteristics of parents (n=98)*		
Age (years)		
21–25	9.1	9
26–35	48.5	48
36–45	33.3	33
46–55	5.1	5
Prefer not to say	2.0	2
Gender		
Female	78.6	77
Male	21.4	21
Educational status		
High school	9.1	9
College	28.3	28
University	33.3	33
Masters	13.1	13
Professional	4.0	4
Doctorate	6.0	6
Other	1.0	1
Prefer not to say	3.0	3
Annual household income		
<£25 000	35.4	35
£25 001–£40 000	21.2	21
£40 001–£80 000	31.2	31
>£80 000	8.1	8
Prefer not to say	16.2	16
Where would you go first if your child had a fever?		
Pharmacy	14.1	14
Walk in centre	14.1	14
General practitioner	37.4	37
NHS 111*	25.2	25
Emergency department	2.0	2
None of the above	5.1	5
*Characteristics of children*		
Age of youngest child
<1 year	38.3	38
1–3 years	34.4	34
4–6 years	12.1	12
7–10 years	12.1	12
11+ years	1.0	1
Age of oldest child		
<1 year	24.2%	24
1–3 years	23.3	23
4–6 years	21.2	21
7–10 years	15.2	15
11+ years	14.1	14
Number of children		
1	47.5	47
2	35.4	35
3	11.1	11
4	0.0	0
5+	2.0	2
Last time any of your children had a fever?		
<3 months	14.1	14
3–6 months	14.1	14
7–12 months	37.4	37
1–2 years	25.2	25
2+ years	2.0	2
None of the above	5.1	5

*NHS 111 is a telephone service for if you have an urgent medical problem and you are unsure what to do.

NHS, National Health Service.

**Table 3:** Characteristics of HCPs completing the DCE

Characteristics of healthcare professionals (n=99)	Percentage	Number
Age (years)
21–25	8.1	8
26–35	57.6	57
36–45	20.2	20
46–55	11.1	11
56+	3	3
Prefer not to say	0.0	0
Years of experience as a HCP		
<5 years	41.4	41
6–10 years	28.3	28
11–15 years	14.1	14
16–20 years	7.1	7
21+ years	9.1	9
Experience working with children		
<5 years	43.4	43
6–10 years	25.3	25
11–15 years	14.1	14
16–20 years	8.1	8
21+ years	9.1	9
Clinical grade		
Healthcare assistant	10.1	10
Staff nurse	28.3	28
Senior staff nurse/Sister	19.2	19
ST1/2	12.1	12
ST3/4	23.2	23
Advanced nurse practitioner	4	4
Consultant	3	3

DCE, discrete-choice experiment; HCP, healthcare provider.

### Parental and HCP preferences for the management of febrile illness

In the DCE, 5/6 attributes for parents and 6/6 attributes for HCPs were statistically significant, suggesting importance with respect to the management of paediatric febrile illness. Table 4 illustrates preferences for each characteristic. Pain/discomfort associated with investigations, and total time in the ED were associated with significant dissatisfaction in both the parental and HCP groups. For HCPs, providing a POC test during triage, which may provide diagnostic information earlier, was associated with significantly increased satisfaction with care. Parents exhibited no preferences for receiving antibiotics, suggesting this is not a meaningful influencer of satisfaction with care in this group; however, for HCPs, a high likelihood of receiving antibiotics was associated with significant disutility. Finally, treatment by doctors in postgraduate training reduced satisfaction with care among both the HCP and parent groups.

**Table 4:** Preferences in the management of paediatric febrile illness of parents and HCP

	Parents (n=98)		HCPs (n=99)	
	Coefficient	95% CI	Coefficient	95% CI
Staff grade				
Trainee doctor	–0.244*	–0.472 to –0.016	–0.204*	–0.398 to –0.099
Nurse practitioner	–0.135	–0.368 to 0.098	0.081*	–0.106 to 0.27
Consultant (reference group)	0.379		0.032	
Likelihood of receiving antibiotics				
Low (reference group)	0.143		0.729	
Medium	0.031	–0.865 to 0.803	–0.111	–0.594 to 0.371
High	–0.174	–0.74 to 0.392	–0.618*	–1 to –0.236
Moderate pain from investigations (relative to low)	–0.462*	–0.613 to –0.312	–0.439*	–0.558 to –0.32
Receive POC test during triage (relative to no)	0.627*	0.484 to 0.769	0.723*	0.562 to 0.884
Total time spent in the ED (per hour)	–0.608*	–0.78 to –0.435	–0.679*	–0.81 to –0.548
Out-of-pocket cost to parents (per £1)	–0.036*	–0.065 to –0.007	–0.051*	–0.074 to –0.028
Observations	2772		2774	
Log likelihood	–722.1		–674.8	

*Significant at 5% level. Table represents β coefficients and CIs from mixed logit regression. The regression coefficients for each attribute level represents the mean part-worth utility of that attribute level in the respondent sample. A positive value denotes utility/satisfaction, with a negative value denoting disutility/dissatisfaction.

ED, emergency department; HCP, healthcare provider; POC, point-of-care.

### Differences in parents’ and HCP’s preferences for the management of paediatric febrile illness

**Figure 1:**
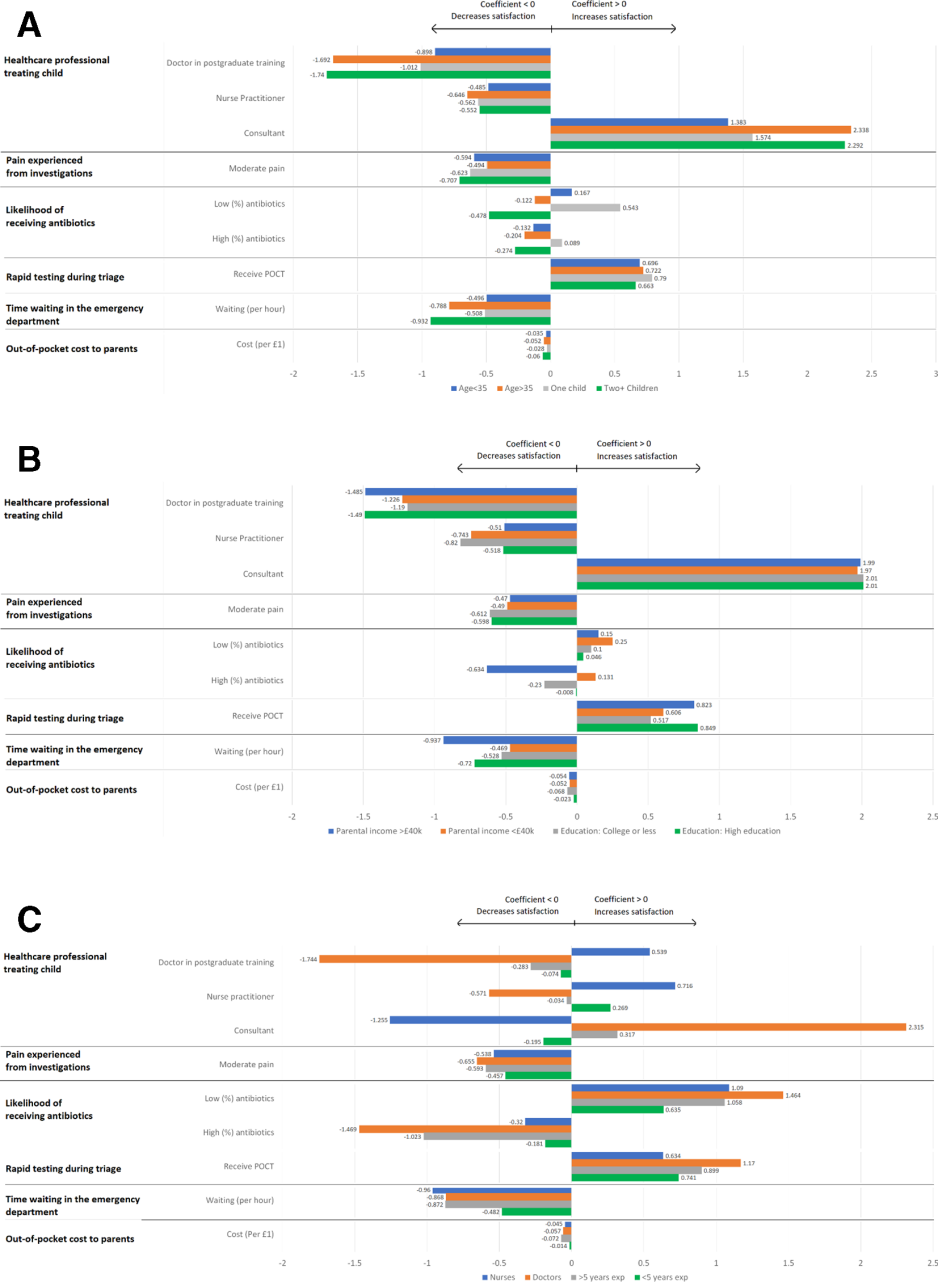
Variation in parents’ (A, B) and healthcare providers (C) preferences for the management of paediatric febrile illness, by subgroup.

Reducing pain from investigations was important among all parent and HCP groups, as was receiving a rapid test during triage. Parents with >1 child and those aged >35 displayed significantly stronger preferences for minimising visit time and receiving consultant-led care, than those with fewer children and those aged <35, as demonstrated in figure 1A. Parents educated to college level or less were less concerned about being managed by a doctor in postgraduate training than those having completed higher education. A moderate/high probability of receiving antibiotics reduced satisfaction among those educated to University level or higher, or with a household income of >£40 000 per year, yet among those educated to college level or less, or with a household income of <£40 000 per year, receiving antibiotics did not affect utility, as shown in figure 1B. All HCP subgroups preferred not to prescribe antibiotics, but none more so than doctors, who also exhibited a stronger preference for rapid-testing than nurses (figure 1C).

### Trade-offs: willingness-to-pay and willingness-to-wait

Parents were willing-to-pay £16.89 (95% CI £8.30 to £26.88) for a 1 hour reduction in total visit time, and £12.83 (95% CI £8.61 to £17.05) to avoid pain from diagnostic investigations. Parents were also WTP £6.77 (95% CI (–) £0.37 to £10.71) to see a consultant, if the alternative was management by a doctor in postgraduate training. Parents expressed a WTW an additional 45.6 min (95% CI (–)19.3 min to 60.4 min) to avoid pain from investigations and 24.1 min (95% CI (–)15.9 min to 46.9 min) for management by a consultant. HCPs were willing to extend waiting times by 39.9 min (95% CI (–)30.9 min to 79.5 min), provided it reduced the likelihood of prescribing antibiotics.

## Discussion

In this first-of-its-kind study, we found that parents and HCPs agree regarding what matters during the management of paediatric febrile illness, a finding which provides reassurance when considering the future implementation and acceptability of novel diagnostics within EDs. Both groups were most concerned about reducing ED visit time, receiving diagnostic information faster and avoiding pain from investigations. The strength of this preference was similar across subgroups of differing sociodemographic characteristics. Parents also displayed strong preferences for being treated by consultants, rather than doctors in postgraduate training. Finally, the likelihood of receiving antibiotics did not significantly influence satisfaction among parents, whereas for HCPs, this was a significant concern. Because the availability of diagnostics is increasing, with CRP-POC testing now used in some UK primary care settings,^21 22^ the findings of this study may be used to prioritise the implementation of upcoming diagnostics, to best meet the preferences of families and HCPs.

A systematic review of emergency medicine highlighted the most frequently identified that interpersonal skills/staff attitudes; provision of information/explanation and perceived waiting times^15^ are most closely associated with parental satisfaction with care. It is likely that as clinical experience increases, so too does confidence in decision making, meaning HCPs can provide greater reassurance, which along with parents equating experience with clinical acumen, may explain why consultant-led care was preferable. This may, however, have some important implications for the implementation of upcoming diagnostics, which may direct low-acuity children to lesser experienced staff, as confidence in diagnostic processes increases, and with this, the seeking of second opinions from more experienced members of staff decreasing.

We identified a strong aversion to children experiencing pain from investigations. While observational data suggest the likelihood of venepuncture during the management of paediatric febrile illness is low,^17^ pain from procedures including venepuncture is often the most traumatic experience when a child’s primary symptom is fever, impacting patient experience significantly.^23 24^ Additionally, studies demonstrate that parents tend to overestimate pain experienced by their children,^25–27^ and therefore our findings suggest that while pain from venepuncture may be expected to last a few minutes, pain from obtaining a single drop of blood from a finger prick for POC testing may be more favourable, thereby improving the experiences for both parents and children.

While substantial literature regarding the management of febrile illness suggests antibiotics are commonly sought by parents,^28–31^ we did not observe this. HCPs demonstrated a strong preference for avoiding antibiotic use where possible, likely a result of increased awareness of the growing threat of antimicrobial resistance; however, parents were indifferent to antibiotic use. This may be explained in part by increased efforts to educate the general population, with television programmes such as ‘Trust me I’m a doctor’, and Public Health England’s ‘keep antibiotics working’ jingle^32^ being just two examples. As such, any novel diagnostics which provide diagnostic information within the window in which precautionary antibiotics are usually considered, are likely to improve HCP satisfaction and patient outcomes, resulting from reduced antimicrobial resistance.

The strengths of our study include the in-depth process for determining attributes of importance, the variety of subgroup analyses performed, and that this study is a first-of-its-kind in measuring preferences for the management of paediatric febrile illness. The findings of this study should, however, also be viewed in the context of several limitations. First, our parent population were sought from the community including playgroups, sure-start centres and parent-teacher associations, rather than those presenting to the ED with fever. While this may be considered a strength in the context of government funded healthcare systems, as the public effectively pays for the National Health Service, this may have affected the accuracy of our results due to recall bias. Second, the sample sizes in the parental and HCP DCEs were limited, which makes robust, precise conclusions, particularly among subgroups, difficult, while the generalisability of our findings may also be limited by all respondents residing in the UK. It is possible that preferences for the attributes considered may differ in other healthcare settings; this was not accounted for in our analysis. Finally, while every effort was made to ensure that the attributes chosen were important to parents and HCPs alike, we could not include every important variable, and as such, it is possible that factors which are influential in determining satisfaction with care were omitted, an issue which future research should aim to address.

## Conclusion

This is the first DCE conducted with parents and HCPs on the choice processes of managing febrile children in the ED. Parents and HCPs feel strongly about reduction of visit time, avoidance of pain and faster diagnosis in the context of managing paediatric febrile illness but are willing to trade these off against each other. Overdue advances in diagnostic capabilities should improve child and carer experience and HCP satisfaction considerably, thus facilitating widespread acceptance and adoption of these technologies.
