# Supplementary material for: What matters when managing childhood fever in the emergency department? A discrete-choice experiment comparing the preferences of parents and healthcare professionals in the UK
Source: Arch Dis Child. 2020 Feb 27;105(8):765–71. doi: 10.1136/archdischild-2019-318209 (PMC7392496; doi:10.1136/archdischild-2019-318209)
Supplement: Supplementary data [file archdischild-2019-318209supp001.pdf]

Supplementary Table 1: Results of coin-ranking exercise

All	Total	Mean	Median	Max	Min
Reducing the amount of time waiting for updates about your child	616	26.8	25	75	0
Spending less time in A&E	393	17.1	20	48	0
Having lots of tests to rule out different causes	322	14.0	12	40	0
Being seen by a more experienced doctor	314	13.7	10	42	0
Minimising pain/discomfort due to tests	300	13.0	12	32	0
Not having to stay overnight in the hospital	149	6.5	0	25	0
Being given antibiotics	119	5.2	5	20	0
