# Supplementary material for: What matters when managing childhood fever in the emergency department? A discrete-choice experiment comparing the preferences of parents and healthcare professionals in the UK
Source: Arch Dis Child. 2020 Feb 27;105(8):765–71. doi: 10.1136/archdischild-2019-318209 (PMC7392496; doi:10.1136/archdischild-2019-318209)
Supplement: Supplementary data [file archdischild-2019-318209supp002.pdf]

1

Question 1/16	OPTION A	OPTION B
TREATING YOUR CHILD	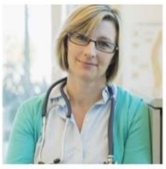 CONSULTANT	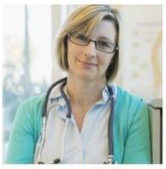 CONSULTANT
PAIN OR DISCOMFORT FROM TESTS	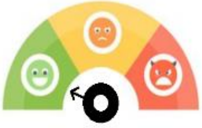 LOW	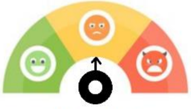 MODERATE
CHANCE OF GETTING ANTIBIOTICS	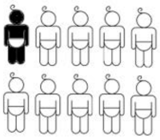 LOW	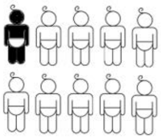 LOW
PERSONAL COST TO YOU	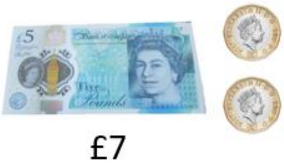 £7	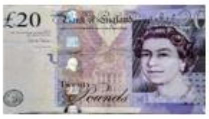 £20
TOTAL TIME SPENT IN EMERGENCY DEPARTMENT	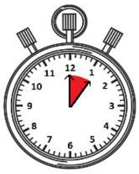 1 HOUR	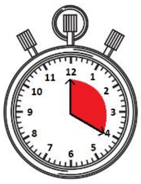 4 HOURS
RECEIVE A QUICK TEST DURING TRIAGE?	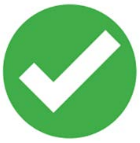	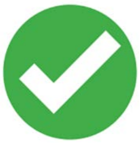
**I CHOOSE …**		

Question 2/16	OPTION A	OPTION B
TREATING YOUR CHILD	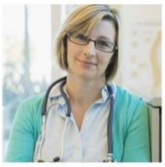 CONSULTANT	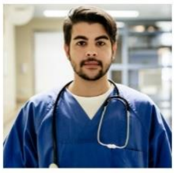 JUNIOR DOCTOR
PAIN OR DISCOMFORT FROM TESTS	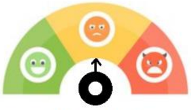 MODERATE	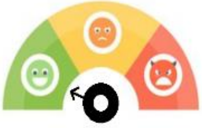 LOW
CHANCE OF GETTING ANTIBIOTICS	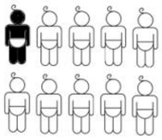 LOW	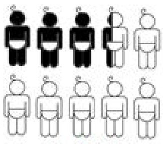 HIGH
PERSONAL COST TO YOU	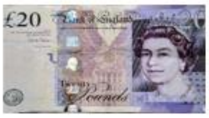 £20	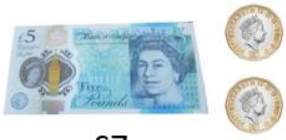 £7
TOTAL TIME SPENT IN EMERGENCY DEPARTMENT	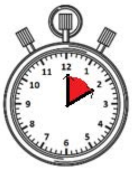 2 HOURS	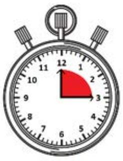 3 HOURS
RECEIVE A QUICK TEST DURING TRIAGE?	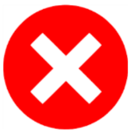	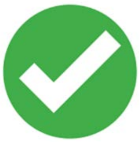
I CHOOSE …	☐	☐

Question 3/16	OPTION A	OPTION B
TREATING YOUR CHILD	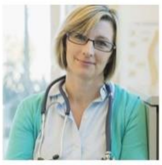 CONSULTANT	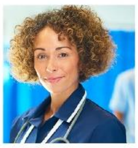 NURSE PRACTITIONER
PAIN OR DISCOMFORT FROM TESTS	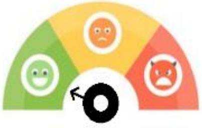 LOW	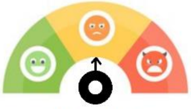 MODERATE
CHANCE OF GETTING ANTIBIOTICS	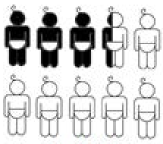 HIGH	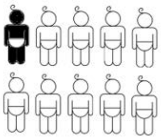 LOW
PERSONAL COST TO YOU	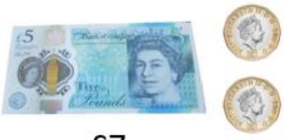 £7	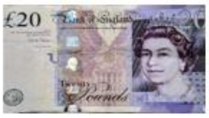 £20
TOTAL TIME SPENT IN EMERGENCY DEPARTMENT	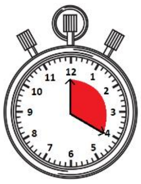 4 HOURS	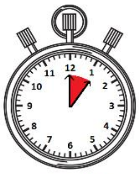 1 HOUR
RECEIVE A QUICK TEST DURING TRIAGE?	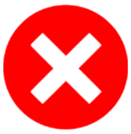	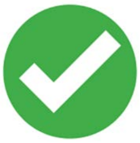
**I CHOOSE …**	☐	☐

Question 4/16	OPTION A	OPTION B
TREATING YOUR CHILD	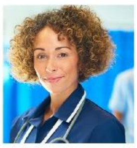 NURSE PRACTITIONER	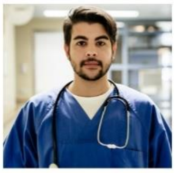 JUNIOR DOCTOR
PAIN OR DISCOMFORT FROM TESTS	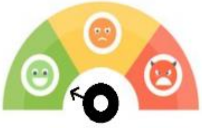 LOW	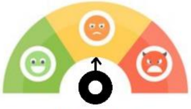 MODERATE
CHANCE OF GETTING ANTIBIOTICS	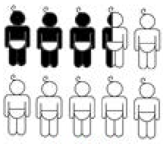 HIGH	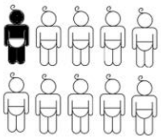 LOW
PERSONAL COST TO YOU	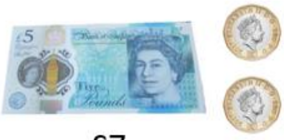 £7	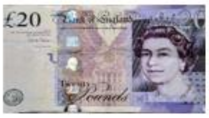 £20
TOTAL TIME SPENT IN EMERGENCY DEPARTMENT	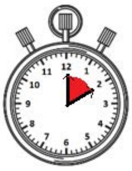 2 HOURS	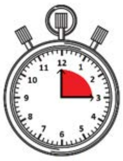 3 HOURS
RECEIVE A QUICK TEST DURING TRIAGE?	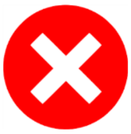	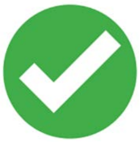
**I CHOOSE …**		

Question 5/16	OPTION A	OPTION B
TREATING YOUR CHILD	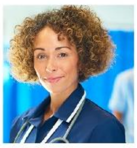 NURSE PRACTITIONER	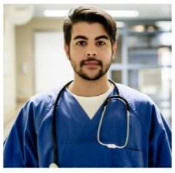 JUNIOR DOCTOR
PAIN OR DISCOMFORT FROM TESTS	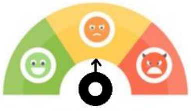 MODERATE	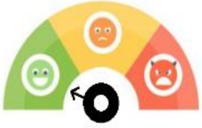 LOW
CHANCE OF GETTING ANTIBIOTICS	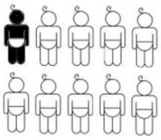 LOW	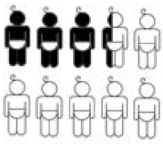 HIGH
PERSONAL COST TO YOU	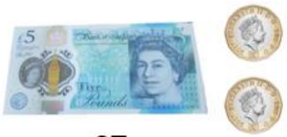 £7	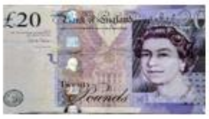 £20
TOTAL TIME SPENT IN EMERGENCY DEPARTMENT	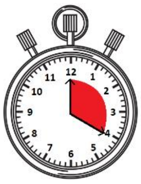 4 HOURS	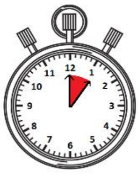 1 HOUR
RECEIVE A QUICK TEST DURING TRIAGE?	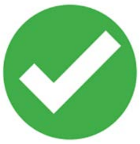	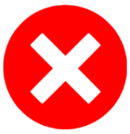
I CHOOSE …	☐	☐

1

Question 6/16	OPTION A	OPTION B
**TREATING YOUR CHILD**	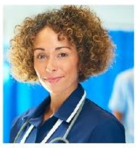 NURSE PRACTITIONER	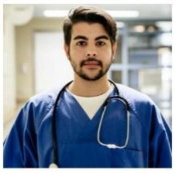 JUNIOR DOCTOR
**PAIN OR DISCOMFORT FROM TESTS**	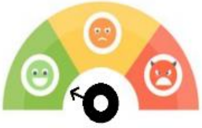 LOW	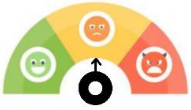 MODERATE
**CHANCE OF GETTING ANTIBIOTICS**	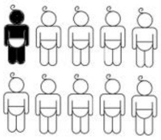 LOW	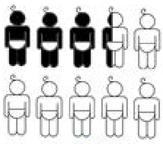 HIGH
**PERSONAL COST TO YOU**	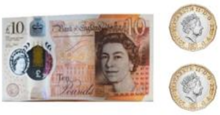 £12	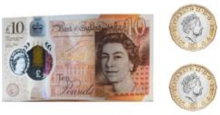 £12
**TOTAL TIME SPENT IN EMERGENCY DEPARTMENT**	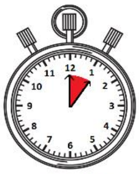 1 HOUR	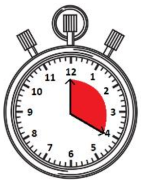 4 HOURS
**RECEIVE A QUICK TEST DURING TRIAGE?**	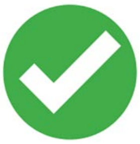	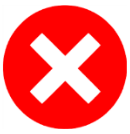
**I CHOOSE …**		

1

Question 7/16	OPTION A	OPTION B
TREATING YOUR CHILD	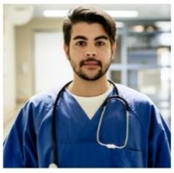 JUNIOR DOCTOR	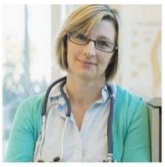 CONSULTANT
PAIN OR DISCOMFORT FROM TESTS	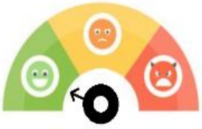 LOW	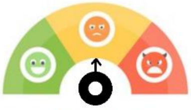 MODERATE
CHANCE OF GETTING ANTIBIOTICS	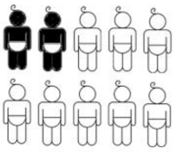 MIDDLE	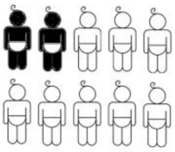 MIDDLE
PERSONAL COST TO YOU	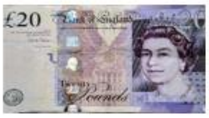 £20	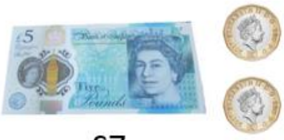 £7
TOTAL TIME SPENT IN EMERGENCY DEPARTMENT	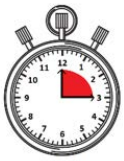 3 HOURS	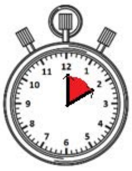 2 HOURS
RECEIVE QUICK TEST DURING TRIAGE?	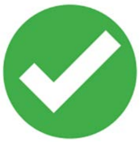	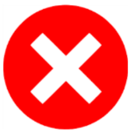
I CHOOSE …	☐	☐

1

Question 8/16	OPTION A	OPTION B
TREATING YOUR CHILD	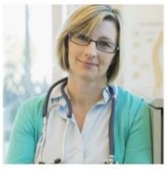 CONSULTANT	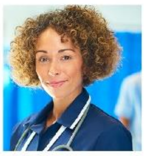 NURSE PRACTITIONER
PAIN OR DISCOMFORT FROM TESTS	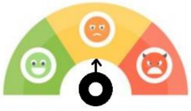 MODERATE	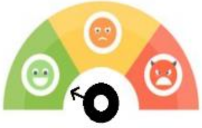 LOW
CHANCE OF GETTING ANTIBIOTICS	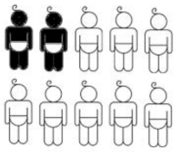 MIDDLE	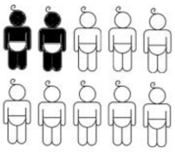 MIDDLE
PERSONAL COST TO YOU	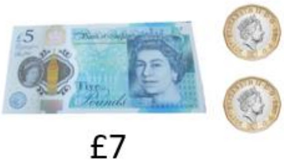 £7	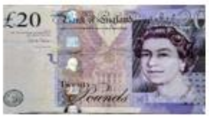 £20
TOTAL TIME SPENT IN EMERGENCY DEPARTMENT	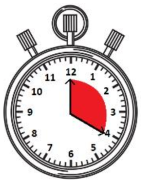 4 HOURS	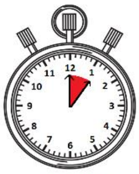 1 HOUR
RECEIVE A QUICK TEST DURING TRIAGE?	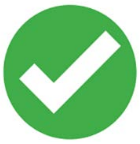	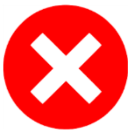
**I CHOOSE …**	☐ ☐	☐ ☐

1

Question 9/16	OPTION A	OPTION B
TREATING YOUR CHILD	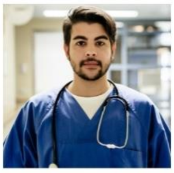 JUNIOR DOCTOR	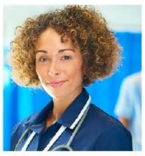 NURSE PRACTITIONER
PAIN OR DISCOMFORT FROM TESTS	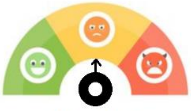 MODERATE	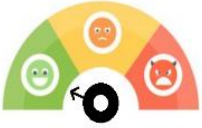 LOW
CHANCE OF GETTING ANTIBIOTICS	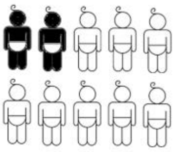 MIDDLE	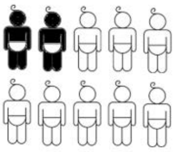 MIDDLE
PERSONAL COST TO YOU	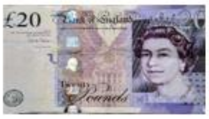 £20	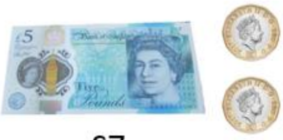 £7
TOTAL TIME SPENT IN EMERGENCY DEPARTMENT	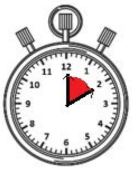 2 HOURS	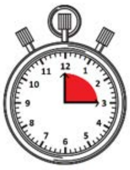 3 HOURS
RECEIVE A QUICK TEST DURING TRIAGE?	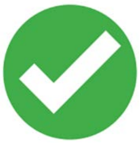	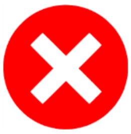
**I CHOOSE …**		

1

Question 10/16	OPTION A	OPTION B
TREATING YOUR CHILD	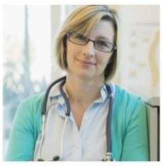 CONSULTANT	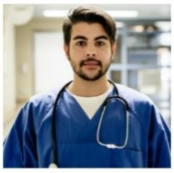 JUNIOR DOCTOR
PAIN OR DISCOMFORT FROM TESTS	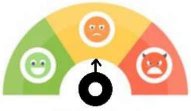 MODERATE	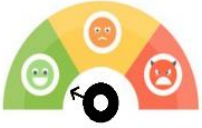 LOW
CHANCE OF GETTING ANTIBIOTICS	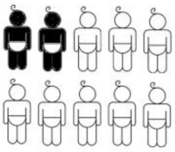 MIDDLE	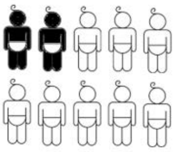 MIDDLE
PERSONAL COST TO YOU	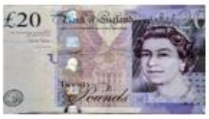 £20	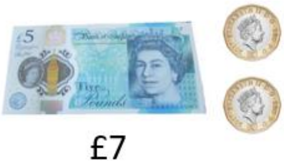 £7
TOTAL TIME SPENT IN EMERGENCY DEPARTMENT	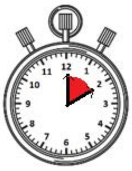 2 HOURS	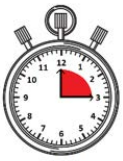 3 HOURS
RECEIVE A QUICK TEST DURING TRIAGE?	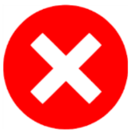	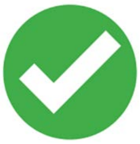
I CHOOSE …	☐	☐

1

Question 11/16	OPTION A	OPTION B
TREATING YOUR CHILD	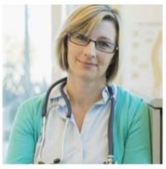 CONSULTANT	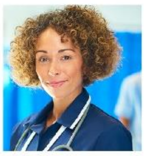 NURSE PRACTITIONER
PAIN OR DISCOMFORT FROM TESTS	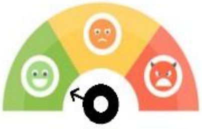 LOW	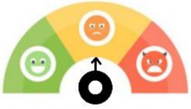 MODERATE
CHANCE OF GETTING ANTIBIOTICS	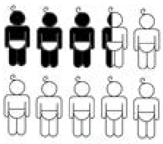 HIGH	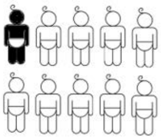 LOW
PERSONAL COST TO YOU	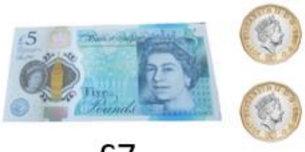 £7	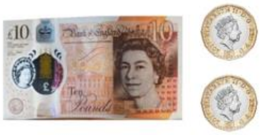 £12
TOTAL TIME SPENT IN EMERGENCY DEPARTMENT	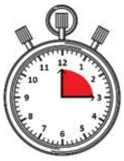 3 HOURS	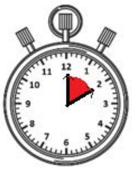 2 HOURS
RECEIVE A QUICK TEST DURING TRIAGE?	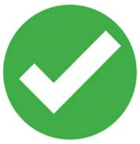	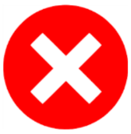
I CHOOSE …	☐	☐

1

Question 12/16	OPTION A	OPTION B
TREATING YOUR CHILD	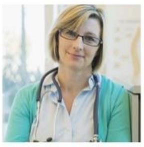 CONSULTANT	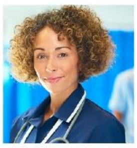 NURSE PRACTITIONER
PAIN OR DISCOMFORT FROM TESTS	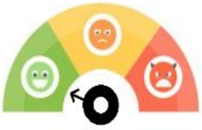 LOW	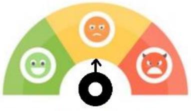 MODERATE
CHANCE OF GETTING ANTIBIOTICS	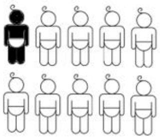 LOW	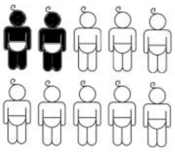 MIDDLE
PERSONAL COST TO YOU	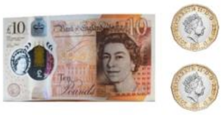 £12	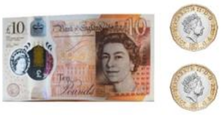 £12
TOTAL TIME SPENT IN EMERGENCY DEPARTMENT	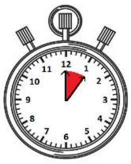 1 HOUR	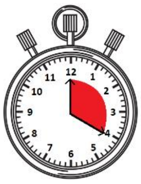 4 HOURS
RECEIVE A QUICK TEST DURING TRIAGE?	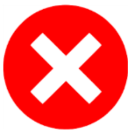	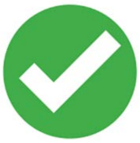
I CHOOSE …	☐	☐

1

Question 13/16	OPTION A	OPTION B
TREATING YOUR CHILD	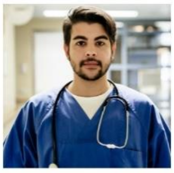 JUNIOR DOCTOR	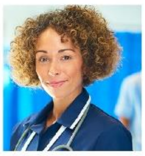 NURSE PRACTITIONER
PAIN OR DISCOMFORT FROM TESTS	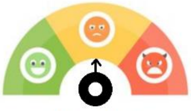 MODERATE	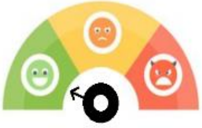 LOW
CHANCE OF GETTING ANTIBIOTICS	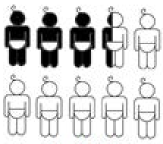 HIGH	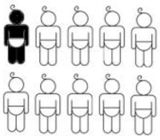 LOW
PERSONAL COST TO YOU	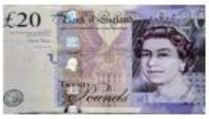 £20	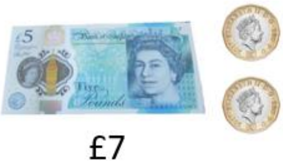 £7
TOTAL TIME SPENT IN EMERGENCY DEPARTMENT	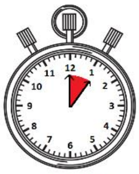 1 HOUR	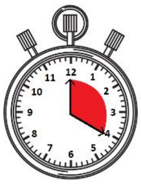 4 HOURS
RECEIVE A QUICK TEST DURING TRIAGE?	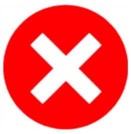	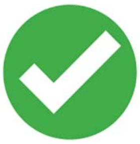
I CHOOSE …	☐	☐

Question 14/16	OPTION A	OPTION B
TREATING YOUR CHILD	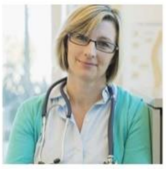 CONSULTANT	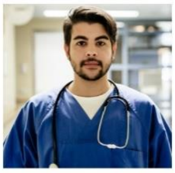 JUNIOR DOCTOR
PAIN OR DISCOMFORT FROM TESTS	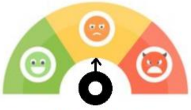 MODERATE	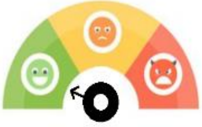 LOW
CHANCE OF GETTING ANTIBIOTICS	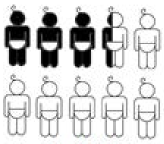 HIGH	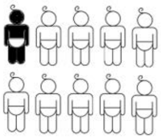 LOW
PERSONAL COST TO YOU	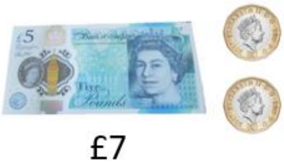 £7	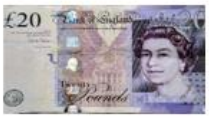 £20
TOTAL TIME SPENT IN EMERGENCY DEPARTMENT	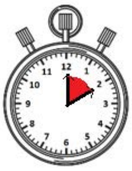 2 HOURS	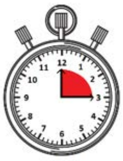 3 HOURS
RECEIVE A QUICK TEST DURING TRIAGE?	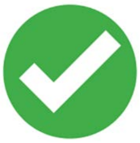	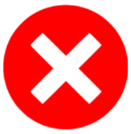
I CHOOSE …	☐	☐

Question 15/16	OPTION A	OPTION B
**TREATING YOUR CHILD**	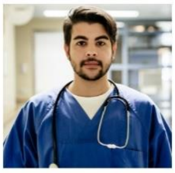 JUNIOR DOCTOR	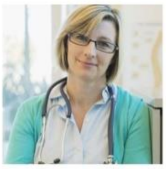 CONSULTANT
**PAIN OR DISCOMFORT FROM TESTS**	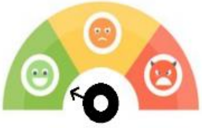 LOW	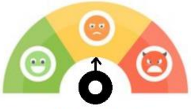 MODERATE
**CHANCE OF GETTING ANTIBIOTICS**	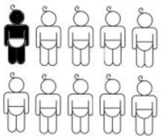 LOW	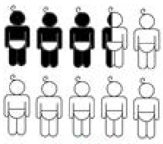 HIGH
**PERSONAL COST TO YOU**	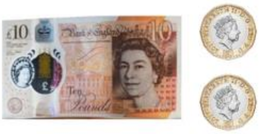 £12	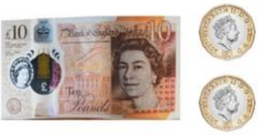 £12
**TOTAL TIME SPENT IN EMERGENCY DEPARTMENT**	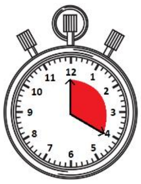 4 HOURS	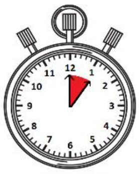 1 HOUR
**RECEIVE A QUICK TEST DURING TRIAGE?**	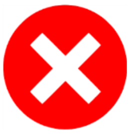	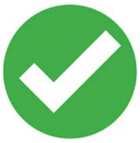
**I CHOOSE …**	☐	☐

1

Question 16/16	OPTION A	OPTION B
TREATING YOUR CHILD	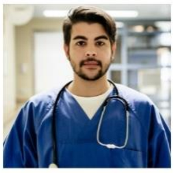 JUNIOR DOCTOR	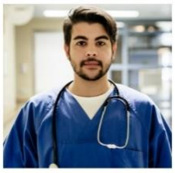 JUNIOR DOCTOR
PAIN OR DISCOMFORT FROM TESTS	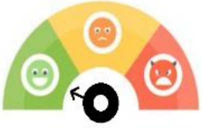 LOW	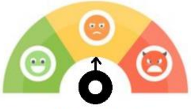 MODERATE
CHANCE OF GETTING ANTIBIOTICS	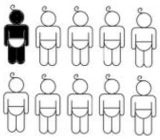 LOW	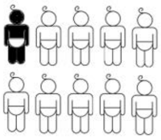 LOW
PERSONAL COST TO YOU	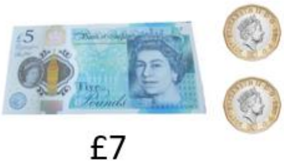 £7	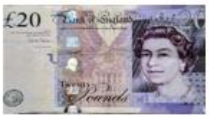 £20
TOTAL TIME SPENT IN EMERGENCY DEPARTMENT	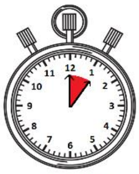 1 HOUR	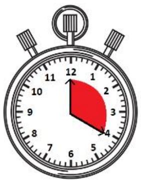 4 HOURS
RECEIVE A QUICK TEST DURING TRIAGE?	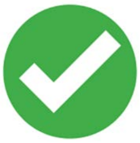	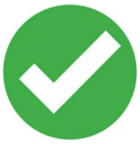
**I CHOOSE …**		
