# Supplementary material for: What matters when managing childhood fever in the emergency department? A discrete-choice experiment comparing the preferences of parents and healthcare professionals in the UK
Source: Arch Dis Child. 2020 Feb 27;105(8):765–71. doi: 10.1136/archdischild-2019-318209 (PMC7392496; doi:10.1136/archdischild-2019-318209)
Supplement: Supplementary data [file archdischild-2019-318209supp003.pdf]

SUPPLEMENTARY TEXT – EXPLANATION OF CODING FOR DISCRETE CHOICE EXPERIMENT

In both effects coding and dummy-variable coding, each non-omitted attribute level is assigned a value of 1 when that level is present in the corresponding profile and 0 when another non-omitted level is present in the corresponding profile. The only difference between the two coding methods is related to the coding of the non-omitted levels when the omitted level is present in the profile. With effects coding (also known as deviation contrast, or ANOVA coding) all non-omitted levels are coded as –1 when the omitted level is present. With dummy variable coding, all non-omitted levels are coded as 0 when the omitted level is present. The coefficient on the omitted level of an effects-coded variable can thus be recovered as the negative sum of the coefficients on the non-omitted levels of that attribute. Therefore, effects coding yields a unique coefficient for each attribute level included in the study, and therefore guarantees that preference weights for all of the levels of a categorical variable sum to zero. This was developed out of the desire to test all category means as deviations against one overall mean value, or the 'grand mean' of all values under consideration. By doing so one avoids preselecting a (potentially arbitrary) reference category as in dummy coding.
