# Supplementary figures and images for: What matters when managing childhood fever in the emergency department? A discrete-choice experiment comparing the preferences of parents and healthcare professionals in the UK
Source: Arch Dis Child. 2020 Feb 27;105(8):765–71. doi: 10.1136/archdischild-2019-318209 (PMC7392496; doi:10.1136/archdischild-2019-318209)

Supplementary Figure 2: Flow diagram of study stages

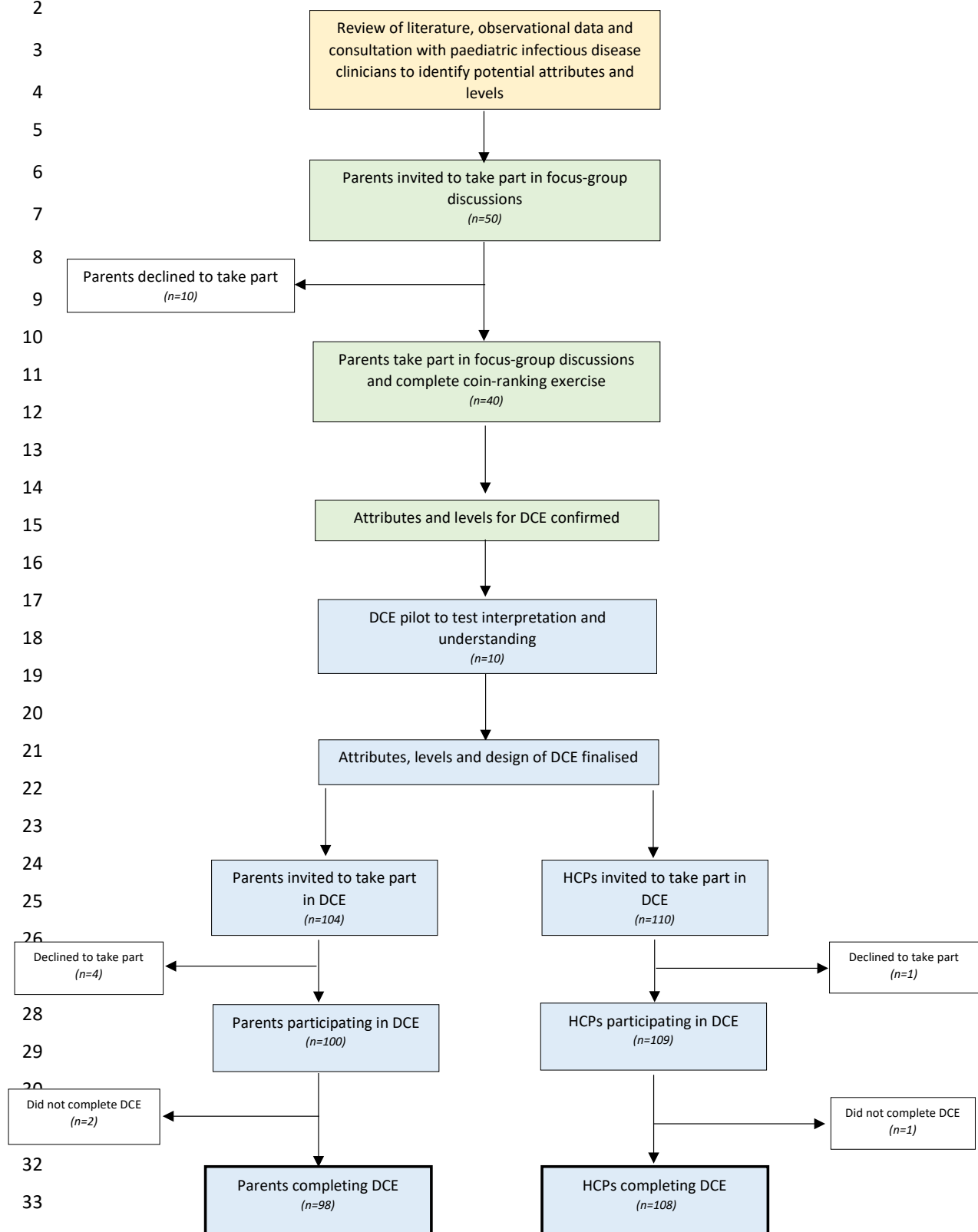

Supplement: Supplementary data [file archdischild-2019-318209supp004.pdf]
